# Co-designing a methodology for workforce development during the personalisation of allied health service funding for people with disability in Australia

**DOI:** 10.1186/s12913-021-06711-x

**Published:** 2021-07-10

**Authors:** Kristen Foley, Stacie Attrill, Chris Brebner

**Affiliations:** 1 Flinders University, Adelaide, Australia; 2 University of Adelaide, Adelaide, Australia

**Keywords:** Workforce, Program logic modelling, Workforce development methodology, Partnership, Allied health professions, Allied health education, Personalisation, NDIS, Disability services

## Abstract

**Background:**

Internationally, health and social services are undergoing creative and extensive redesign to meet population demands with rationed budgets. This has critical implications for the health workforces that serve such populations. Within the workforce literature, few approaches are described that enable workforce development for health professions in the service contexts that emerge from large scale service redesign in times of industry shift. We contribute an innovative and robust methodology for workforce development that was co-designed by stakeholders in allied health during the personalisation of disability funding in Australia (the introduction of the National Disability Insurance Scheme).

**Methods:**

In the context of a broad action research project, we used program logic modelling to identify and enact opportunities for sustainable allied health education and workforce integration amidst the changed service provision context. We engaged with 49 industry stakeholders across 92 research engagements that included interviews (*n* = 43), a workshop explicitly for model development (*n* = 8) and a Project Advisory Group (*n* = 15). Data from these activities were inductively coded, analysed, and triangulated against each other. During the program logic modelling workshop, we worked with involved stakeholders to develop a conceptual model which could be used to guide trial and evaluation of allied health education which was fit-for-purpose to emerging workforce requirements.

**Results:**

Stakeholder interviews showed that drivers of workforce design during industry shift were that (1) service provision was happening in turbulent times; (2) new concerns around skills and professional engagement were unfolding for AHP in the NDIS; and (3) impacts to AHP education were being experienced. The conceptual model we co-designed directly accounted for these contextual features by highlighting five underpinning principles that should inform methodologies for workforce development and AHP education in the transforming landscape: being (1) pedagogically sound; (2) person- or family-centred; (3) NDIS compliant; (4) informed by evidence and (5) having quality for all. We use a case study to illustrate how the co-designed conceptual model stimulated agility and flexibility in workforce and service redesign.

**Conclusions:**

Proactive and situated education of the emerging workforce during policy shift is essential to realise future health workforces that can appropriately and effectively service populations under a variety of changing service and funding structures – as well as their transitions. We argue that collaborative program logic modelling in partnership with key stakeholders including existing workforce can be useful for broad purposes of workforce (re)design in diverse contexts.

**Supplementary Information:**

The online version contains supplementary material available at 10.1186/s12913-021-06711-x.

## What is known about the topic

1. Health workforces must be equipped to provide appropriate and effective services under a range of policy and industry structures
2. Funding structure, and the shift between funding structures, plays a key role in scaffolding opportunities for workforce development
3. There are limited methodologies proposed for workforce development generally, as well as a dearth of those that address contexts of funding transition and/or allied health

## What this study adds

1. An innovative co-design process for workforce development, drawing on partnership program logic modelling with industry stakeholders
2. Contextual evidence regarding the key drivers of workforce design for allied health professionals during the large scale shifts associated with personalised funding
3. Modelling of how such contextual evidence can be synthesised into strategies to ensure the ongoing provision of a sufficient and skilled health workforce in new service structures.

## Introduction

Adept and flexible workforces are required to meet health and social service needs of complex populations. In times of economic rationalisation, policy and industry shift are not uncommon as governments around the world work to appropriately and effectively distribute these workforces. The personalisation of funding for disability services is a primary example of this, with an array of countries in Europe, Britain, South America and the Middle East transitioning to individual funding models. Policy underpinning these schemes intends for consumers to procure services that fit their needs, assuming that this will result in service provision that responds to consumer wants and needs over the long term [1–4]. New opportunities for service provision emerge, while others require redesign to respond to consumer-driven demands as they unfold in the market to remain financially viable. Workforce/s that can effectively recognise and service the demands of the consumer base are therefore essential to the successful translation and realisation of such policy change.

Yet, despite the critical and extensive nature of workforce (re)consideration and (re)design necessary to realise these policy changes, limited literature addresses how workforce development can be enabled in the service contexts that emerge from marketisation. Limited methodologies are evident that support processes of workforce design in times of industry shift [5]. Generally, workforce design tends to be reactive rather than proactive [6–8]. Mismatches in the demand, supply and affordability of service provision – key workforce concerns – are known to be major barriers to innovation in healthcare [9]. High administrative burdens accompany policy and service redesign on the ground [10]. These phenomena can obstruct service providers from being able to engage with processes that build and equip future workforce/s – both current and emerging [11, 12].

We consider the emerging workforce to be central to general discussion about workforce because in many health disciplines their training is firmly integrated with service provision, where providers play a crucial role in workplace-based training and education. During times of major policy and industry shift, such as marketisation, the multiple important and urgent demands on service providers can foreclose their engagement with the essential tasks of supervision, placement coordination and mentoring. At the same time, the emerging workforce may require skills that are still under development by their hosting service provider – a new rendering of ‘traditional’ (centrally-funded) clinical practice techniques in ‘market’ settings where the consumer is highly engaged in planning [13, 14]. These specific challenges for education and training of the emerging workforce further complicate the transition of the broader workforce into new policy environments.

The marketisation of disability services is a productive environment to examine issues of workforce development, because a wide range of professional disciplines service this population in addition to an extensive largely unregistered workforce. In Australia, the health and disability sectors were relatively distinct until the recent transition to personalised funding for people with disability and aged care through the introduction of the National Disability Insurance Scheme (NDIS) and My Aged Care respectively, both legislated in 2013 [15]. Allied health professionals (AHPs) in Australia work quite differently in the different sectors. In the disability sector they work with people with disabilities to assist decision making regarding supports that enable choice and control in how they might set and achieve personal life goals. In the health sector, they work with people to assess and manage both acute and chronic health problems. The shifts in funding therefore present novel and fragmentary contexts in which health and social care workforces must respond to emerging requirements for skills, capacity and cross-sectoral planning in line with philosophical change and policy requirements. A critical example of this are the thin markets which have emerged in rural and remote areas of Australia, where the government used to be the main service provider. Consumer held funding has driven increased demand for allied health services in these areas but also nationally. With a very small existing rural and remote workforce that already experiences transcience, the high demand and competition for workforce in metropolitan centres presents significant challenges for equitable access to services.

In this paper we describe a methodology for workforce development during times of industry/policy shift. We argue that collaborative program logic modelling can support AHPs more broadly to map existing and emerging workforce needs in transitional and diverse service contexts – in complex workforce landscapes undergoing change. The landscape of the NDIS transition, and our focus on allied health workplace education, serve as contextual features for the development of our methodology – as all workforce changes have their own nuances. We make three contributions to the evidence base: showcasing (1) the drivers of service and workforce design during the South Australian experience of transition to personalised disability funding; (2) a methodology for workforce development that was co-designed with industry stakeholders in a turbulent service context; and (3) a conceptual model that served as an output of the program logic modelling process.

## Methods

Herein, we describe the setting of our project, the co-design approach we followed and how program logic modelling was used as a methodology for workforce design that accounted for transitioning service, industry and policy environment/s for which current and emerging workforces must be adequately and proactively prepared.

### Project setting

Our action research project was undertaken in South Australia in response to declining opportunities for workplace allied health professional (AHP) education amidst Australia’s transition from centrally- to personally-controlled funding for disability services through the NDIS. The project’s central concerns were to (1) generate a rich understanding of considerations around AHP education in this new service landscape; (2) use program logic modelling to develop a conceptual model which would guide and evaluate AHP education with stakeholders in the field; (3) trial the utility of the model in live settings with AHP students in diverse service contexts and examine its usefulness in supporting workforce planning; and (4) refine the conceptual model so it can usefully conceptualise new, innovative and sustainable opportunities for AHP education in marketized service landscapes. We employ the term *AHP education* because it encompasses a focus on practice placements as a critical component to ensure that AHP students are work ready, while also enabling flexibility to address the broader architecture in which practice placements are embedded (i.e. curriculum, other university-provider interactions). The project team was comprised of six researcher-practitioners and two placement facilitators, six of whom were speech pathologists and two occupational therapists.

### Co-design approach

The project team took a co-design approach to the project. Co-design has been shown to facilitate innovative, workable solutions to challenging problems through exploring problems whilst co-creating solutions [16]. Well constructed co-designed projects facilitate collaboration and a team approach to the design, evaluation and application of solutions which transform the end-user experience. Program logic modelling as an approach aligns well with the co-design framework, because it can be undertaken collaboratively to explicate and explore potential solutions. Thus, a wide variety of industry stakeholders were integrated throughout the project, contributing to the methodology through diverse, collaborative activities. The stakeholders involved are detailed below in Table 1. Across the project, there were 49 stakeholders from a range of areas including allied health professionals to bureaucrats, and eight research team members (*n* = 57 individuals in total). Of these, 13 stakeholders participated in multiple project activities and other stakeholders’ involvement varied across the project depending on the stage and focus. Altogether, excluding participation by researchers, there were 92 research engagements throughout the project that contributed to the workforce development methodology. AHPs were represented from the disciplines of Speech-Language Pathology, Occupational Therapy and Physiotherapy.

**Table 1:** Key stakeholder involvement

Stakeholder group	Number involved	Participant Identifier	Number of project engagements
Allied Health Educators	8	AHE1 (PAG-1,4) AHE2 (PAG-1) AHE3 (interview + PAG-1,2 + workshop) AHE4 (interview + PAG-1,3,4 + workshop) AHE5 (interview × 2 + workshop) AHE6 (PAG-1) AHE7 (interview) AHE8 (workshop)	2 1 4 5 3 1 1 1 **18**
Service Providers	17	PROV1 (interview + PAG-1) PROV2 (interview × 2 + workshop) PROV3 (PAG-1,2) PROV4 (interview × 2 + PAG-1,2,3,4) PROV5 (interview × 2) PROV6 (interview × 2) PROV7 (interview) PROV9 (interview) PROV10 (interview) PROV11 (interview) PROV12 (interview) PROV13 (interview) PROV14 (PAG-1,4) PROV15 (workshop) PROV16 (interview) PROV 17 (interview)	2 3 2 6 2 2 1 1 1 1 1 1 2 1 1 1 **28**
Chef Financial Officers	3	CFO1 (interview × 2 + PAG-1,2,3,4 + workshop) CFO2 (interview) CFO3 (interview + PAG-3,4)	7 1 3 **11**
Bureaucrats	5	B1 (PAG-1,2,4) B2 (PAG-1,3) B3 (PAG-1,2,3,4 + workshop) B4 (PAG-1,3) B5 (PAG-3)	3 2 5 2 1 **13**
Disability Advocates	2	DA1 (interview ×2 + workshop) DA2 (interview × 2 + PAG-1,2,4)	3 5 **8**
Students	12	S1-12	**12**
NDIS recipients	2	REC1-2	**2**
Total participants	49*	exclusive of 8 research team	
Total activity involvements	92*	exclusive of engagements by research team	

*Please note these numbers are exclusive of the research team as participants and their contribution to the total activity involvements

### Recruitment

Stakeholders from allied health education, service provision and disability advocacy were recruited through university networks; often known to members of the research team through professional networks. Occasionally, stakeholders suggested another organisation or individual that would provide a valuable contribution to the dataset. The action research methodology enabled us to respond to emerging gaps in our knowledge to recruit new groups of stakeholders who could provide additional information or expertise to inform the context and program logic [17]. For example, Chief Financial Officers were invited to participate when the financial viability of service providers was identified as a key influence on decreasing education opportunities for AHPs following the transition to the marketised environment. Bureaucrats, who executed the NDIS policy and funding through localised government agencies, were recruited through industry networks. Allied health students were recruited through online posts to forums for courses in which they were enrolled. Students were all from the same institution, and were either in their final or penultimate year of study. Finally, NDIS recipients were recruited through service providers participating in the project.

### Ethics

Full ethical approval was provided by Flinders University (project number 7551). This included five approved modifications throughout the course of the project to enable the project and research activities to respond to the developing context or research limitations (i.e. purposively sampling for provider diversity in later stages of the project, or modifying the recruitment strategies used to recruit NDIS recipients).

### Program logic modelling

Program logic modelling aims to explicate the ‘logic’ by which a program should achieve particular outcomes in a given context – it is the theory behind how a particular program should work (see for example [18]). The central purpose of program logic modelling is to map out the assumed logic of how the mechanisms of a program will lead to expected outcomes in particular context/s [19, 20] – attempting to predict causal chains of influence in the real world [21]. By using this approach in a co-design framework, we enhanced the opportunities for co-creation of innovative solutions to the workforce problem. Thus we utilised program logic modelling collaboratively with stakeholders in AHP service provision to map the contextual influences on AHP education during policy transition, to develop a project (i.e. program) that ‘would work’ appropriately and effectively to enhance opportunities for educating the emerging AHP workforce in live contexts of service delivery. Exploring and understanding the working context experienced by service providers due to policy and industry change associated with the NDIS was a specific aim of program logic modelling.

**Fig. 1:**
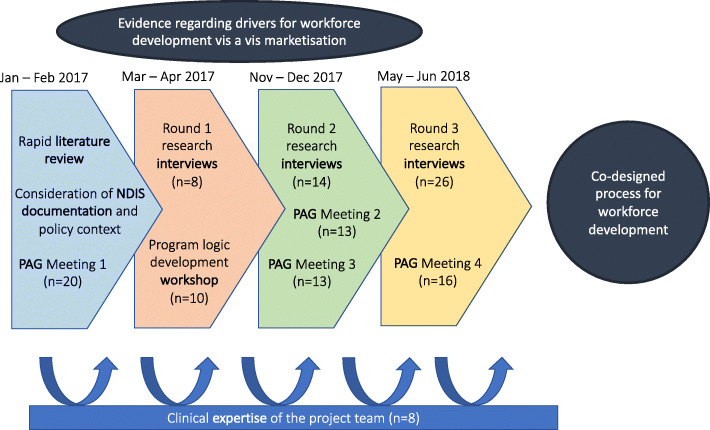
Data sources which informed development of program logic modelling and co-design of workforce development. (Please note that PAG refers to Project Advisory Group, who are described later in the article)

There were a variety of data inputs to the program logic modelling, outlined in Fig. 1.

A major driver of the logic model construction was a program logic model workshop. To inform this activity, the project team initially collected data from stakeholders during eight interviews to generate contextual evidence about the drivers for service provision and AHP education during marketization. This evidence was presented to stakeholders at the beginning of the program logic modelling workshop, with the logic model developing via consideration of what the project Outcomes should be, achievable via which Mechanisms (represented as strategies in the conceptual model) in the given Context (therefore identifying the anticipated CMOs of the project, foundational to program logic modelling). A conceptual model was produced through the program logic modelling process, which was then used to facilitate the project. It was also refined during the project, as it was trialled in its efficacy to support the redesign of workplace education opportunities for emerging AHP workforces. There were therefore iterative waves of data collection throughout our project, which all had a role in informing the final version of the program logic and conceptual model – as shown in Fig. 1. The waves of data collection are described in turn below.

#### Rapid review of the grey literature

A review of grey literature available on workforce development and the NDIS in Australia was undertaken in early 2017 and enabled us to understand known barriers and enablers to workforce development processes across a range of health/social care disciplines (*n* = 8 sources; i.e. government reports from South Australia, Tasmania, New South Wales, Victoria and Queensland regarding how health workforces could/should prepare for the NDIS, position statement from Australian Health Professions Australia). Please contact the corresponding author for copies of the documents included in this review.

Examples of foundation concepts drawn from this review were: consumer choice and control in NDIS services; the benefits students can bring to service providers during times of industry change; and the complexities in developing new points of interface between sectors (i.e. health, education, justice) as a result of policy change.

#### NDIS documents and policy context

Policy documents related to the rationale for the introduction of the NDIS, the funding specifications and implementation processes were analysed to examine the guiding principles articulated to underpin the policy change. Releases from the National Disability Insurance Agency (NDIA) were tracked to keep abreast of how the scheme was unfolding and how the administration of the scheme was being adjusted iteratively to meet the needs of NDIS participants and service providers. Examples of the documents can be found at the project website that emerged from the project (www.openlearning.com/courses/NDIS).

#### Project advisory group

A Project Advisory Group (PAG) was established at the commencement of the project, and data derived from group meetings was included in the analysis. The Terms of Reference for the group are included as Additional file 1. The PAG was convened on four occasions throughout the project – first, early in the project to contribute insight on the emerging industry context and known tensions with workforce development; second and third, at two midway points through the project to review and provide feedback on the research findings about the emerging industry context and the conceptual model; and finally, during the end stage of the project to reflect on the usefulness of the project as a methodology to guide the identification and resolution of workforce needs. The PAG meetings were transcribed by a member of the project team, with minutes circulated amongst the group to confirm their fidelity.

Recruitment for the PAG followed a network-based and purposive approach, where potential members were invited based on their portfolio and role in AHP service provision and/or education. As a result, the PAG was represented by 15 individuals from a diverse range of disability service providers, government agencies and universities, and included a variety of expertise and roles. One of the PAG members retired between meetings two and three, and was replaced by the incumbent in her role for meetings three and four.

#### Model development workshop

The research team facilitated a workshop to map the context, mechanisms and planned outcomes of how to increase opportunities for AHP education in marketized service settings – i.e. to draft the program logic, which aimed to produce a conceptual model to guide the project activities and outcomes. This 3 h face to face workshop was facilitated at a central Flinders University campus by one member of the research team, who firstly outlined existing knowledge about the context (drawn from the rapid review, NDIS documentation, first PAG meeting, first round of interviews (*n* = 8) and clinical expertise). The nine stakeholders that attended the workshop co-constructed a conceptual model with the seven research team members (total *n* = 16) to identify opportunities for AHP education which ‘would work’ for participating services amidst the changing service provision landscape. During the workshop, the research team encouraged participants to draw from practice examples with consumers to illustrate whether or not the sketched logic was valid in their services. At the end of the workshop the program logic was drafted into a conceptual model and agreed on by the attending stakeholders. Photos of the visual mapping processes were captured to ensure the positioning of logic links and model elements were retained when later translated to a computer-generated image of the model. The workshop was live-transcribed by a member of the project team, and the transcript circulated to the project team for verification.

#### Stakeholder research interviews

The research team developed semi-structured interview guides to explore topics germane to each stakeholder group. The guides were drawn from the rapid literature review and document analysis, and were iteratively adjusted to respond to themes identified during the project. In Rounds 1 and 2, service providers and allied health educators were interviewed about how the workforce had changed or adjusted in response to the NDIS transition, what workforce gaps had become evident, and what opportunities there were for allied health student practice placements to achieve appropriate and effective education outcomes in the changed service provision environment. In Rounds 2 and 3, these stakeholders were asked about the usefulness of the conceptual model in designing and refining student placements. NDIS recipients were asked what workforce they wanted to meet their goals and needs. Student interviews explored their experiences of placement education in live settings of NDIS service provision. Chief Financial Officers were asked about new and old processes for business planning and about perspectives on financial viability, and changes to AHP role/s in these activities.

Interviews ranged from 25 to 45 min. For all stakeholder groups except students, interviews were conducted one-on-one. Students were offered the option of group interviews, and five students were interviewed across two group interviews, with the remaining seven students completing one-on-one telephone interviews. For the other stakeholders (*n* = 31 interviews), 22 were conducted in person and nine via telephone, according to what the stakeholders reported was convenient and requested. All interviews were audio-recorded and transcribed verbatim by a professional transcription company.

#### Clinical expertise of the project team

Each of the AHP team members brought their understanding of AHP service provision and the marketizing transition to the project, and made collaborative decisions throughout the project about the next steps of the project informed by data generated through the project. Weekly meetings were held with the project team to discuss research findings, administrative and ethical requirements, and the next steps for progressing the project.

### Data analysis and interpretation

The immediate purpose of data analysis during the project was to collate emerging knowledge generated during data collection activities and use this to plan what to do next within the project. All data were first inductively analysed by Author 1 and presented to the project team during weekly meetings for input and refinement. This initial analysis provided a coding framework (shown in Table 2) for later deductive analysis that assisted in refining the conceptual model.

**Table 2:** Coding framework generated during inductive analysis

Need for Quality but Constraints. High Focus on Financial Viability and Billability. Multiple Competing Priorities – Before/Coinciding with Hosting AHP Education. Need for an Effective and Appropriate Workforce. Flux and Flurry – Turbulent Landscape of Service Provision.	

The initial themes were refined as the project progressed, and whilst these were elaborated on and deepened, they remained largely unchanged throughout the project. Any inductive codes that were outside of the coding framework were recorded and discussed at subsequent coding meetings. The ideas built through these iterative processes provided an axis for the research team to reflect about the earlier coding and interpretation (i.e. abductive inference following [22]), and helped the team to identify and verify any presences/absences that were not a focus during the first stages of coding and analysis. These deductive processes verified the model as it was applied in practice.

### Rigour

The stakeholders involved in our project were purposively sampled to engage multiple and diverse vantage points that helped to identify different assumptions and knowledge(s) around how the program should work [21, 23]. This enhanced our ability to capture complexity and breadth of perspectives and context to inform the logic to be embedded within the model. The complexity, cohesion and iterative development of the research findings was further evidenced by the participation of thirteen allied health educator and service provider stakeholders, in multiple activities. These stakeholders provided continuity across project activities that enhanced the development, implementation and review of the model. Our combination of inductive and deductive inquiry throughout the research helped interrogate findings and key assumptions against each other [22].

The project team carefully considered the knowledge that was generated at each data collection stage before deciding what should happen next in the project. These considerations were interpreted through the team’s clinical and education expertise that included contextually relevant practice areas including paediatric and adult disability, public and community health, organisational management and policy, clinical education and tertiary education leadership. This meant the research team entered and directed the project already holding significant experience in relevant fields. As Berger [24] outlines, there are both challenges and benefits to researcher familiarity with the research topic. Employing reflexivity was critical to ensure that the project team didn’t reconstitute their own understanding of workforce gaps in the NDIS, and to examine bias from their own perceptions [24] of processes and practice related to consumer-controlled funding. Fortnightly team meetings were held to purposefully discuss potential assumptions and views of each researcher that might be influencing the interpretation of findings and planning of the next steps. Presentation of inductive data was a feature of these meetings, to support researchers to remain reflexively attuned to the insight emerging from stakeholder participants through the dataset.

## Findings

**Fig. 2:**
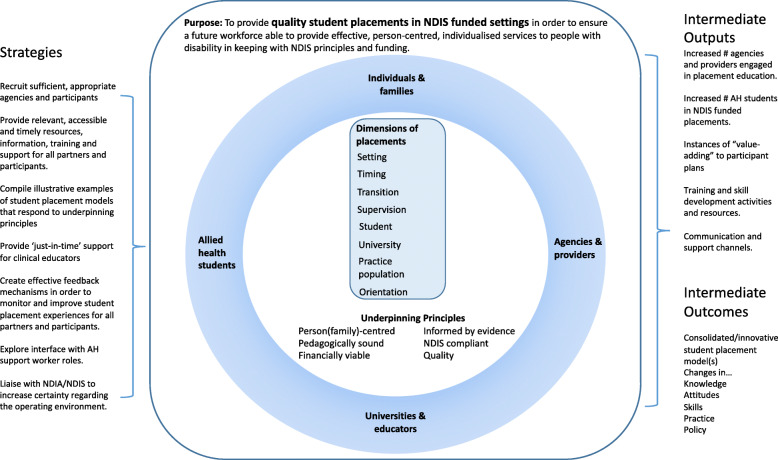
The conceptual model

We present the key issues identified early in our dataset, predominantly through interviews, that formed the context of our collaborative program logic modelling. These findings form the backdrop of our workforce development methodology and were included in the conceptual model we present in Fig. 2 as underlying principles. We present these findings here to provide insight to the nuanced arena in which workforce development must operate – and how this knowledge was embedded into the conceptual model. Beyond ensuring that our workforce development methodology accounted for the emergent landscape of service provision and industry change, our findings can also be taken alone as evidence about how service providers conceptualise AHP education during a time of policy change and turbulent service provision. We use three headings to report the background findings for the program logic modelling: (1) service provision in turbulent times; (2) AHP and the NDIS: new concerns around skills and professional engagement; and (3) impact/s on AHP education.

### Service provision in turbulent times

Stakeholders flagged that providing services at the time of the NDIS rollout was challenging. New billing, compliance and administrative procedures were required to apply for and obtain payment from the funding agency. Further to needing to develop new procedures, service providers also needed to revise these responsively as the rollout of the Scheme progressed in order to function effectively.

“It is a massive system that is forever changing. The requirements and guidelines are unclear and are challenging for therapists that are working in that system to keep up to date with and understand because of all of the inconsistencies that occur.” *[Chief Financial Officer #1]*

Providers described that these new procedures were effortful for AHPs. While some organisations did have dedicated management, finance and administrative staff, most AHPs were directly implicated in enacting new administration and billing procedures because they worked at the interface with the recipient and had to tailor and report on their service in line with pre-identified therapeutic goals.

“The [AHP] students have also been involved in trying to understand the budgets as well… we’ve showed them how we will look at their budget to work out if [the NDIS recipient has] got any funds available.” *[Service Provider #2]*

In addition to administrative and billing procedures, providers reported that who applied for or received services, and the nature of the services requested were changing as NDIS recipients were enacting choice and control of their services. Additionally, some previous services were no longer financially viable in the new model, or no billable time existed for activities previously completed for clients.

“… for funding of groups, we thought this was something we could get students to work in with, but it’s still $52 an hour for a group, but in terms of cost recovery it’s not viable for us to do that. There is a lot of education we need to provide to the NDIA about what’s valuable versus viable.” *[Service Provider #14]*

In some cases individuals or families were obtaining services for the first time. Some providers identified that this diversified the clinical practice they offered to recipients, because families who hadn’t previously received services had entered the system. Providers identified additional time provided by AHPs to support families to navigate the system or respond to challenges in obtaining payment. This time was not recognised as a billable activity through the NDIS.

“So many families that are coming in for their initial meetings are being encouraged to self-manage… and they’re not using the money to pay for our services.” *[Service Provider #6]*

The influx of new families stimulated demand for AHP services. All providers identified challenges in recruiting AHPs to appropriately service this demand, so many organisations had long waiting lists. Managing these waitlists, increasing service capacity, and redesigning services became central concerns for NDIS providers during and following rollout.

### AHP and the NDIS: new concerns around skills and professional engagement

In addition to the administrative changes, stakeholders identified that AHPs were required to promote their services and use a service co-design approach with consumers, which had not been necessary under centrally-controlled funding. This was explicitly articulated as a new skillset for AHPs, requiring the mapping of services according to consumer demand and personalisation to achieve the individualised services intended by the Scheme. AHPs required time and practice to develop these new skills.

“… one issue we’re having at the moment is really just… that change to a program which has been operating for such a long time and changing to [NDIS recipients] having the choice about what they want to do and where they want to go…” *[Service Provider #5]*

Despite recognising the need to develop these skills, providers reported they had less time to dedicate to these tasks due to the high waitlists, effort to build the skills of new families to navigate the system, and flux in AHPs moving between organisations. Stakeholders identified that limited time existed to undertake mentoring, professional development or specialisation because it was not directly billable, and that this applied to students placed with them as well as early career practitioners. Service providers pointed to the ‘certain amount of claimable hours’ enclosing service interaction/s and execution of related activities.

“… as much as the client work that the students are doing would be – it would be claimable time in some way, every staff member here who is providing NDIS service has KPIs to meet. We have a certain amount of claimable hours that we need to do in a week, and that at the moment doesn’t leave a lot of wriggle room for extra admin and extra supervision, like support for people.” *[Service Provider #1]*

Many service providers expressed concern that the need to prioritise billable activities – the focus on KPIs – also reduced time/capacity to engage in professional development. In turn, providers linked this with reduced opportunities to proactively develop their own or junior practitioner clinical skills, and was a central point of concern around how ‘best practice’ service transitioned into a marketized scheme. Disability advocates identified that clinical governance processes that directed service administration often remained unchanged from previous central funding models. This was reported to lead to conflict related to how NDIS recipients were included in decisions regarding their services and interventions, including how they were enabled to make choices that contrasted with practitioner values about ‘best services’ or how to accommodate consumer wants and needs that were not offered within the service.

“…[NDIS recipients would] say, I want more. More is best. They weren’t really provided with any support or information about the fact that more wasn't necessarily best. So people have ended up many examples of families saying, I want 20 sessions of speech, 20 sessions of OT, 20 sessions of physio. I don't want to pay for collaboration, communication, co-ordination, that's not valuable. I just want more of you seeing - sitting in front of face-to-face with my child.” *[Disability Advocate #2]*

### Impact on AHP education

All stakeholders emphasised that AHP education opportunities should exposed students to the turbulent and consumer-directed nature of service provision. They reasoned that this provided a foundation for the future workforce to develop the skills, approaches and resilience needed for effective and adequate practice.

“… it’s really important to train the next group of students coming on, because how else are we going to get quality service provision if the students don’t – if the students don’t get it. If they come out of a four year degree and with no – none of that culture of working with people with disabilities… understanding the complexities and the range of supports that you might provide, then well they’re not going to choose to come into this sector which means that our clients are not going to get adequate services.” *[Disability Advocate #1]*

Despite this, however, service providers reported significant barriers in accepting AHP students for workplace education. They reported that engaging with students in placements required them to balance time and energy judiciously across student and service activities to ensure they continued to meet billable activities targets. Financial considerations that related to the business of the service were prioritised over extraneous, non-billable activities, including those of AHP education or workforce development.

“… [student education] comes at a cost for the practice. In an NDIA world there is no mechanism there to be able to help or level out the extra financial burdens for the practice or burdens for the clinician that’s taking those students on… If we’re taking a chunk out of [our resources] to put into students that – we’re also removing that chunk and that capacity from our practice and from the core work that we would be doing.” *[Chief Financial Officer #1]*

A further issue was a lack of transparency about how student-led services may be billable under NDIS funding structures. Service providers identified challenges in establishing clarity with NDIA around these, and other administrative issues.

### Setting the context for program logic modelling

The above themes were discussed and refined with stakeholders who attended the model development workshop, and then populated as the ‘context’ section of the program logic. These themes therefore provided the basis for collaborative development of the program logic, because (after refinement from key stakeholders) they outlined the *context* in which AHP workforce development, AHP education and concomitant service provision must take place. The next steps to map the *outcomes* and *mechanisms* of the project resulted in a conceptual model, which we present in Fig. 2.

A series of ‘underpinning principles’ were developed out of the findings to ensure that the knowledge about the context in which workforce development must operate was accounted for in the conceptual model – the program logic. The underpinning principles and their connection with the themes presented above are shown below in Table 3. These principles were developed through the collaborative approach with the stakeholders involve in student placements in new workplace environments. Co-designing the principles with these stakeholders enabled a partnership approach to AHP education that met all stakeholder needs, therefore transforming the user experience, and reflecting a substantially different process that is normally undertaken by universities in facilitating AHP workplace education.

**Table 3:** Crafting Underpinning Principles that responded to the Program Logic Context

Underpinning Principle	Rationale and Relation to Theme/s
Pedagogically sound	Authentic, quality education experiences are required, situated within the service provision landscape, that appropriately prepare AHP students for future NDIS market needs and service provision
Quality for All	Maintains central focus on quality in AHP education as well as service provision, vis a vis major changes in the service landscape and wider industry
Person (Family)-Centred	Encapsulates the key intentions of the NDIS, and therefore the driver of service re-design – whether NDIS recipients are enabled to enact choice and control
NDIS Compliant	Necessary foundation for service providers to engage with NDIA and NDIS recipients, reflecting the extensive work providers are already undertaking to achieve this
Informed by Evidence	Links with concerns regarding the erosion of governance processes; highlights the importance of professional development and collaborative stakeholder engagement

### Using program logic modelling to develop a conceptual model

Once the context for the program logic had been modelled (and translated into underpinning principles), we shifted to co-designing the project *outcomes* and *mechanisms* with stakeholders. The mechanisms are shown in the conceptual model as strategies which lead to tangible outputs to reach the overarching project outcomes and purpose – thereby mapping the anticipated logic of the project. The outcomes of the project were drafted in relation to providing quality placement-based education as a way of ensuring a future workforce, and were agreed by stakeholders to focus on long-term changes to skills and knowledge of AHPs in alignment to transforming service provision. Rather than identifying either mechanisms or outcomes in isolation, their development was an iterative process of brainstorming potential steps, and then using co-design principles to work back and forth through conversation with stakeholders to ensure there was logical integration between the planned strategies and outcomes (which retained awareness of the context in which these strategies and outcomes would occur).

The conceptual model is presented below in Fig. 2, and can be considered in two ways: firstly, as an output of the process to co-design a workforce design methodology; and secondly, as a visual representation of the methodology for workforce development itself. In our case, it guided the remainder of the project by keeping everyone accountable to the planned outcomes and mechanisms for workforce development in the emergent context.

We believe this conceptual model has broad utility for stimulating and supporting (re)consideration of workforce, beyond the specific application of engaging with AHP education. The ‘dimensions of placements’ box, which we explain shortly, could be adapted to represent context-specific details that will have a practical influence on how workforce development must proceed. Drawing from our specific focus on AHP education during the project, we describe how the conceptual model functioned with stakeholders to explore new and adapted practices for AHP education in a turbulent and transitioning service provision context.

### Using the model to identify and enact new opportunities for AHP education

**Fig. 3:**
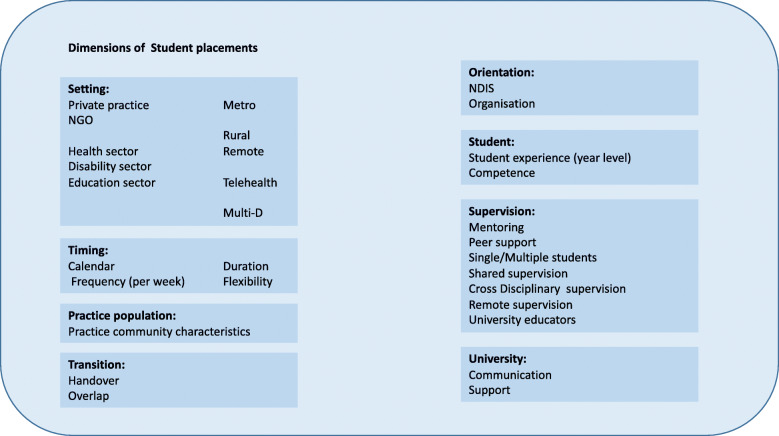
Considerations of student placements and service provision raised during the program logic modelling process

During the program logic modelling workshop, specific ‘dimensions of placements’ were raised by stakeholders as relevant and important levers on placement based workplace AHP education. Including these data points in the conceptual model helped to link the broad considerations around the workforce development context and the project outcomes for workforce development, to the specific requirements for structuring AHP education in each particular workplace. We could not include all of the detail in the visual representation of the model, so showcase the specifics here in Fig. 3.

We now provide a case study to illustrate the value of the conceptual model (and methodology) for addressing issues of workforce design. As part of our action-research project we employed two placement facilitators to work with service providers to explore new opportunities for AHP education through practice placements. The conceptual model became integral in this work, as a way of visual brainstorming – identifying new opportunities for AHP education brought about because of the changed locus of choice and control over services – now residing with consumers. Their work with a particular service provider during the project provides a useful case study, reported in Table 4 to illustrate the utility of the conceptual model, and by extension the co-designed workforce development methodology.

**Table 4:** Case Study Exemplar of Model In-Situ.

LilaCARE* is a not-for-profit community organisation who provides comprehensive services to adults following brain injury. The shift to consumer-directed funding, via the NDIS, most significantly impacted their Community Rehabilitation and Transition* (CRT) program. The CRT supported adults with brain injury to transition back to the community following a period of subacute inpatient rehabilitation. Multiple AHP disciplines were involved to enact these transitions, and prior to the enactment of the policy change, LilaCARE had regularly hosted AHP students on placements. The CRT was identified to be unsuitable as a service provision model in the NDIS landscape due to funding and administrative constraints. Funding for CRT clients had previously been received in 3 year blocks, which was not compatible with the annual funding cycle delivered through the NDIS. Further, it was unclear how the program could be administered using market logic, as service demand was unpredictable and holding recipients on waiting lists would delay client transition into the community. LiliCARE also had several questions about the compliance of supervision activities. For example, it was unclear if practitioners needed to be ‘in the room’ in order to bill for a service activity. Previous centrally-controlled funding was administered at the service provider level, so the provider themselves governed whether students were appropriately skilled and able to provide this service. The status of student-led service activities in relation to billability was not transparent in policy or procedural information that related to the NDIS. Placement facilitators used the model with LilaCARE staff to identify new opportunities for AHP student placement that aligned to the client and service provision needs, and market environment. They explored the **dimensions of student placements**, to elucidate detail of how student placements did or could function within LilaCARE’s service offerings. Examples related to **timing** (services were completed in half day sessions that were incompatible with traditional student placement hours; students arrive in placement blocks), **setting** (LilaCARE has close partnership with health sector and CRT Program involves multidisciplinary work, both of which had to develop new pathways following marketisation), **supervision** (remote, shared and cross-disciplinary supervision was considered to facilitate students to be placed in more environments) and **university** (support and communication from university staff to facilitate new supervision models). A consideration of existing/potential **placement dimensions** were brainstormed to account for novel ways in which LilaCARE could offer student placements. The **underpinning principles** provided a contextual framework for whether the emerging possibilities/ideas for student placements would be fit-for-purpose for the needs of the service provider, and within the NDIS service landscape. This collaborative process facilitated the implementation and trial of new methods for clinical supervision for placement students as **outputs** (i.e. peer and mentor learning, type of supervision levels, cross-disciplinary supervision). Further, a framework was developed that enabled students to lead group sessions, that were a service activity considered less financially viable in the individualised funding environment. To enact these changes, LilaCARE supervised more students who were also from different disciplines. To realise this, novel communication and support structures were developed with **universities.** During the trial of new supervision processes, the placement facilitators worked responsively in partnership with service providers to overcome challenges as these arose – for example, how to navigate constraints on physical space when multiple staff and students were present at the service. The conceptual model was then used as a tool for evaluation with stakeholders. Staff from LilaCARE were interviewed, and asked to use the model to reflect whether/how the adaptations to student placements achieved the **purpose** – *quality placements with NDIS service providers –* and the **underpinning principles***.* LilaCARE reported that the innovations posed **financially viable** and **pedagogically sound** placement opportunities. During evaluation, LilaCARE staff identified a gap in students’ placement preparation, and **university** changes were enacted to ensure students understood how **person-centred** practice is enacted for NDIS recipients. *names have been changed to preserve the confidentiality of the organisation who participated	

The case study is intended to show the program logic modelling process supported the construction of the *context* of AHP service and workforce design during industry shift through to the co-design of *mechanisms* to achieve collaboratively-identified *outcomes.* For LilaCARE, the conceptual model produced a contextual framework to identify how AHP student placements could be included in the service design, and also guided the implementation and evaluation of those placements. Because we engaged multiple stakeholders in mapping the context of AHP student placement education, the logic modelling process was grounded in a variety of different perspectives. This co-design process was mirrored in LilaCARE’s application of the model where stakeholders used the logic collectively to problematize complex issues around AHP student placement activity in context, and then used the underpinning principles to guide the placement trials and evaluation. This case study illuminates how the process of collaborative program logic modelling is a valid and valuable methodology for (re)design of AHP education as a specific feature of workforce development.

## Discussion

Workforces are unlikely to become or remain static in the face of pressing contemporary needs to rationalise health and social services. Continual changes to the work and conditions of a range of workforces (i.e. health, education, disability, aged care) must be expected. Our research, situated in a transitional policy period, illuminates this environment of ‘constant change’ and suggests that it cannot be assumed that the previous workforce will be able to transition or adapt to the new paradigm. Proactive workforce planning is needed to account for service design, capacity, skills and AHP education. The methodology for workforce development developed out of our research can enable processes of workforce (re)consideration and (re)design in and through policy shift. As it stands, workforce development in the health sector tends to be reactive rather than proactive, is sometimes overlooked altogether [6], and often takes a crisis to trigger meaningful action [7]. There is also a tendency to perceive student education as not being ‘core business’, and as negatively impacting productivity, despite education of students being essential for future workforce development [25]. Sparse literature [5] and a lack of consensus on the available literature [26] exists about appropriate methodologies for design of the health workforce. A notable exception is that scholars agree workforce development is complex and that this complexity must be accommodated for in processes of workforce design.

Complexity was a major theme in our research, and the co-design of ‘underpinning principles’ during program logic modelling enabled this complexity to stay in focus throughout the project. A major threat to workplace planning is working in silos to attempt reducing the complexity involved [27]. Workforce initiatives must be ideologically and pragmatically tethered to the complexity of service provision and its related pressures in times of policy shift [28]. Our methodology enabled us to focus on what service providers actually do [29] rather than operating on assumptions or abstract knowledge. The co-design element of our methodology generated a detailed view of organisational and institutional dynamics as they articulate with workforce planning [30], enabling the drivers for workforce intervention to stay connected to practice – thereby enhancing the likelihood of effective intervention [31]. Obtaining a detailed understanding of the complexity as it presents in the workforce problem/s it generates – in our case, declining student placement numbers and increasing demand for AHPs – created more opportunities for innovation [32].

There is a dearth of AHP literature that has explored workforce planning [30] and how AHP education interfaces with workforce design. High workloads and staff shortages have been shown to compromise effective AHP education [33]. Good workforce planning, conversely, enhances AHP retention [34] and may respond to ongoing workforce issues such as the lack of AHPs in regional locations [35]. Evidence-informed research and practice is urgently needed in light of ongoing major health and social care reforms. Building minimum datasets about the AHP workforce is required [12, 36], although this work must attend to the diffuse role boundaries, fractions and scope of practice [26] intrinsic to the allied health professions.

Workforce initiatives can tend to focus on workforce types rather than workforce skills [37], but it is not clear how this will carry in contexts of marketisation. Traditional university-controlled placement planning requires service providers to offer placements that meet education requirements, and do not necessarily attend to the needs of the service or clients. However, our method explicates how working in partnership with and centralising key stakeholders in the process of workforce design develops a shared understanding of the unexpected challenges, shortfalls and opportunities amidst service redesign. In contexts of high workforce pressure and high workforce demand, there are greater needs for workplace support and mentoring [38]. In our research, however, service providers perceived these activities to be at odds with financial survival in the short term. This is of critical concern and reaches beyond education of AHP students. Spending time unpacking the experiences and conflicting priorities of AHPs working in service provision contexts, and operationalising this knowledge to collaboratively develop and implement solutions for AHP education was essential to our workforce design methodology. Collaborating in this way led to flexible supervision and mentoring across professions [39], showcasing the major contributions that inter-professional education can make to workforce development [40, 41].

Our research and co-designed methodology contribute specific value in the emerging context of marketisation, where historical systems of service allocation are not fit for purpose. Rather, complex demand-based models are required [42] that account for what consumers want. A workforce strategy is necessary to ensure staff retention and the assurance of quality amidst workforce change agendas, particularly where productivity becomes derivative [43]. Ensuring the quality and authenticity of the emerging workforce through education is enmeshed in these concerns. Broadly, a paradigm shift is needed to reframe planning of the health and allied health workforces away from a focus on shortages to more effectively organising and educating the workforce to meet the needs of consumers and the broader community [8]. Consciousness-raising, long-term visioning and collaboration are known by-products of program logic modelling [21] – demonstrated through our project – so our methodology may also be useful to this end.

Future research must explore the voice of service recipients in contexts of marketization, and particularly in the context of their access to quality services that meet their needs and wants – to what extent they feel they can participate [14]. While in marketisation consumers tacitly shape service provision offerings, through their purchasing decisions, their voices are not represented in research about AHP workforce planning and there is little concept of what they might want [13]. Adding further complexity to this scenario is that marketised schemes can often spark tensions between needs/wants of consumers and health professional beliefs or values, animated in clashes over power, dominance and authority as schemes of marketisation become embedded [44]. A pertinent example in allied health is that consumers with their own funding may prefer to purchase therapy assistants as well as than AHPs in order to stretch their funding and/or meet workforce shortfalls in particular professions [45, 46]. In some cases this has led AHPs to advocate to consumers their value [47], but it does raise that little is known about the beliefs (and potential conflicts) of consumers about AHP skills and services. In order to appropriately inform AHP education, research must therefore amplify consumer voices in relation to workforce planning and how the workforce can be supported to ensure they have the capabilities to provide services that meet consumer needs.

### Strengths, limitations, and contextual considerations of our research

A major strength of our research was the overlay of participatory action-research with collaborative program logic modelling, because it enabled the integration of stakeholder perspectives directly into the workforce model, co-designing the solution [21, 48]. The action research methodology supported responsiveness to social context throughout the research project to account for knowledge raised during the project, a hallmark of good quality qualitative research [17]. We were also able to maintain contact with our stakeholders throughout the project and engage them in multiple research activities. We argue that their longitudinal engagement with the project was of benefit to the research as it sensitised stakeholders to reflect on their experience over time and provided the opportunity for this new knowledge to be circulated back into the project and research.

We used both inductive and deductive processes of inquiry within our qualitative research, which encouraged us to consider knowledge developed during our project from multiple perspectives [22]. Our sample size (*n* = 49) included stakeholders from different facets of AHP service provision, education, policy and advocacy, which enhanced the breadth of perspectives that informed the development of contextual knowledge within our project. A major limitation of our research was its limited ability to engage the voice of consumers in the process of workforce design (*n* = 2). We have advocated for the importance of this in future research throughout the paper.

There were more Speech Pathologists recruited to our research than other AHPs, and the disciplines available to participate included Speech Pathology, Occupational Therapy and Physiotherapy. Consequently this research cannot be generalised into all AHP contexts. Our research was also conducted in South Australia at a time of early NDIS implementation, which privileged the provision of services for paediatric clients. This means that some of the contextual workforce challenges (i.e. findings and context) reflected in our project may be germane to paediatric work, however testing of the conceptual model in adult disability contexts has not suggested this. Other regions of Australia may have different experiences, because of their service geography or a range of other social/cultural factors, and also because the policy may have been adjusted in response to issues identified during the early implementation phase. Results may also differ now that the policy is further normalised and embedded across Australia.

## Conclusion

Our research points to the value of proactively undertaking workforce design to ensure that the AHP workforce can service consumers appropriately and effectively in times of significant industry and policy change, and we contribute a robust methodology to undertake this important work. Our research highlights that collaborative processes of workforce design generate knowledge about the complexity and real-life experience of policy/service transition and can be purposed to develop opportunities that bolster the current and emerging AHP workforce. The workforce development methodology we have outlined draws program logic modelling to craft a grounded understanding of the complexities of service provision and organisational priorities that envelop issues of workforce development. We propose that this process can support diverse stakeholders to explore innovative opportunities for the AHP workforce during times of intense and profound policy/industry change now and into an uncertain future.

## Supplementary Information

**Additional file 1.**

## Data Availability

Please contact the corresponding author for any queries related to the data and materials utilised to inform this study.
