# Supplementary material for: Co-designing a methodology for workforce development during the personalisation of allied health service funding for people with disability in Australia
Source: BMC Health Serv Res. 2021 Jul 10;21:680. doi: 10.1186/s12913-021-06711-x (PMC8272260; doi:10.1186/s12913-021-06711-x)
Supplement: Supplementary file 1 — Additional file 1. [file 12913_2021_6711_MOESM1_ESM.doc]

## Project Advisory Group Terms of Reference

**Background**

Speech Pathology at Flinders University is conducting a project funded by the Department for Industry and Skills to develop strategies and resources that will sustain ongoing development of an allied health workforce capable of providing quality services to people with a disability in a market-style environment. This project runs until June 2018 and aims to build capacity to provide more student placements in NDIS funded services and to develop a model of service and resources that can be used by all stakeholders to support ongoing growth and development of allied health services in this sector.

This project will develop allied health workforce capacity to deliver NDIS funded services into the future through 5 inter-related strategies:

1. Building the capacity of the existing allied health workforce to respond to user choice and control, provide individualised and accountable person- or family-centred care, and contribute to education of the future workforce.
2. Equipping future allied health workforce to work collaboratively and innovatively with people with disabilities through high quality placement experiences.

In order to achieve these aims the project will:

1. Work in partnership with key stakeholders to explore opportunities and develop financially sustainable model(s) that will incorporate student placements that are NDIS compliant, meet the needs of participants, students and practitioners.
2. Develop multi-disciplinary resources to advance students’ knowledge and skills for provision of quality NDIS funded services to people with disabilities while on placement.
3. Create and disseminate professional development activities, resources and web-based materials to:
   1. Support allied health practitioners and services to engage in quality service delivery for people with disabilities.
   2. Provide high quality placements for students
4. Support students to be prepared to complete placements with NDIS funded clients and services.

**Term**

This Terms of Reference is effective from Wednesday 7 February 2017 until Friday 29 June 2018, or longer as required.

**Project Advisory Group role and responsibilities**

The overall purpose of the Project Advisory Group is to provide a critical review of the project planning, implementation and dissemination and to provide links to stakeholder groups.

Members will be asked to:

- provide advice and direction to the project design and evaluation
- review the Activity Work Plan, including updates as needed
- bring organisational and sector knowledge to the study
- bring broad representation of stakeholder perspectives.

Members will adhere to the project’s ethical requirements; (e.g. individual respondents must not be identified during data analysis and reporting).

# Frequency of meetings

# The Project Advisory Group will meet 2-3 times a year in 2017 and 2018.

**Model development and evaluation overview and timetable**

| **Stage** | **Method** | **Timeline (approx.)** |
| --- | --- | --- |
|  |  |  |
| Stage 1  Identification of barriers and opportunities | Project Advisory Group established  Information gathering/document review of relevant literature, guidelines etc  Interviews with policy, practice, education and consumer stakeholders | Feb-Mar 2017 |
| Stage 2  Collaborative development of NDIS student placement models | Model development workshop(s)  Interviews re early implementation issues with policy, practice and education stakeholders  Feedback to Project Advisory Group | Mar- June 2017 |
|  |  |  |
| Stage 3  Evaluation of trial implementation | Interviews with policy, practice and education stakeholders  Interviews with NDIS service recipients (consumers)  Focus group with students  Routine student placement feedback Model revision workshop  Feedback to Project Advisory Group | June 2017- April 2018 |
| Report/  Dissemination | Includes short plain English summary for participants available via web or post | April – June 2018 |
